# The nurse-led GILL eHealth intervention for improving physical health and lifestyle behaviours in clients with severe mental illness: design of a cluster-randomised controlled trial

**DOI:** 10.1186/s12888-023-05024-z

**Published:** 2023-09-15

**Authors:** Meike M. Hoogervorst, Berno van Meijel, Esther Krijnen-de Bruin, Aartjan Beekman, Nynke Boonstra, Marcel Adriaanse

**Affiliations:** 1 Department of Psychiatry, Amsterdam UMC and Amsterdam Public Health Research Institute, Amsterdam, The Netherlands; 2 Department of Health, Sports and Welfare, Inholland University of Applied Sciences, Amsterdam, The Netherlands; 3 Parnassia Psychiatric Institute, Parnassia Academy, The Hague, The Netherlands; 4 Department of Psychiatry, UMC Utrecht Brain Center, University Medical Center Utrecht, Utrecht, The Netherlands; 5 NHL Stenden, University of Applied Sciences, Leeuwarden, The Netherlands; 6 KieN VIP Mental Health Care Services, Leeuwarden, The Netherlands; 7 Department of Health Sciences, Vrije Universiteit Amsterdam and Amsterdam Public Health Research Institute, Amsterdam, The Netherlands

**Keywords:** Severe mental illness, Somatic screening, eHealth intervention, Lifestyle behaviours, RCT, Process evaluation, Metabolic syndrome

## Abstract

**Background:**

Clients with severe mental illness (SMI) have overall poor physical health. SMI reduces life expectancy by 5–17 years, primarily due to physical comorbidity linked to cardiometabolic risks that are mainly driven by unhealthy lifestyle behaviours. To improve physical health in clients with SMI, key elements are systematic somatic screening and lifestyle promotion. The nurse-led GILL eHealth was developed for somatic screening and the implementation of lifestyle activities in clients with SMI. Aims of this study are to evaluate the effectiveness of the GILL eHealth intervention in clients with SMI compared to usual care, and to evaluate the implementation process, and the experiences of clients and healthcare providers with GILL eHealth.

**Methods:**

The GILL study encompasses a cluster-randomised controlled trial in approximately 20 mental health care facilities in the Netherlands. The randomisation takes place at the team level, assigning clients to the eHealth intervention or the usual care group. The GILL eHealth intervention consists of two complementary modules for somatic screening and lifestyle promotion, resulting in personalised somatic treatment and lifestyle plans. Trained mental health nurses and nurse practitioners will implement the intervention within the multidisciplinary treatment context, and will guide and support the participants in promoting their physical health, including cardiometabolic risk management. Usual care includes treatment as currently delivered, with national guidelines as frame of reference. We aim to include 258 clients with SMI and a BMI of 27 or higher. Primary outcome is the metabolic syndrome severity score. Secondary outcomes are physical health measurements and participants’ reports on physical activity, perceived lifestyle behaviours, quality of life, recovery, psychosocial functioning, and health-related self-efficacy. Measurements will be completed at baseline and at 6 and 12 months. A qualitative process evaluation will be conducted alongside, to evaluate the process of implementation and the experiences of clients and healthcare professionals with GILL eHealth.

**Discussion:**

The GILL eHealth intervention is expected to be more effective than usual care in improving physical health and lifestyle behaviours among clients with SMI. It will also provide important information on implementation of GILL eHealth in mental health care. If proven effective, GILL eHealth offers a clinically useful tool to improve physical health and lifestyle behaviours.

**Trial registration:**

Clinical trial registration NCT05533749, registration date: 8 September 2022.

## Background

Clients with severe mental illness (SMI) have overall poor physical health. SMI reduces life expectancy by 5–17 years [1–3]. The high mortality rate is mostly due to natural causes such as COPD, cancer and cardiometabolic diseases [4, 5], with cardiometabolic risks (e.g. obesity, diabetes, hypertension, dyslipidaemia) as most prominent cause [1, 6]. Clients with SMI are more affected by these cardiometabolic risk factors than the general population [7]. Main reasons for the high prevalence are the adverse effects of psychotropic medications and unhealthy lifestyle behaviours such as smoking, poor diets, and physical inactivity [8–11]. Furthermore, clients receive insufficient monitoring and therapy for cardiovascular and other diseases, and use healthcare facilities and national screening programmes less often than the general population [5, 12].

To improve physical health in clients with SMI, key elements are comprehensive somatic screening and lifestyle promotion [5]. Research on the effects of somatic screening in mental health care is still scarce. More research on lifestyle promotion has been conducted, proving that lifestyle interventions can reduce weight and cardiometabolic risks in persons with SMI [13]. Lifestyle promotion also contributes to improved mental health outcomes in a selection of clients with SMI [14]. Although paying attention to lifestyle promotion is effective and considered essential care, the systematic implementation of both somatic screening and lifestyle interventions appears challenging in current mental health care [15]. To realise a better implementation of systematic screening and lifestyle promotion, multidisciplinary efforts are needed, with mental health nurses and nurse practitioners being in charge as lifestyle care managers [16, 17].

Use of eHealth can significantly contribute to effective implementation of programmes for physical health promotion in mental health care, as advised by experts [5]. eHealth can be defined as health care provided by computers or internet technology, such as websites and mobile device applications. With eHealth clients and mental health professionals can get in touch easily, and clients are enabled to gain more control over their own health and lifestyle behaviours by applying self-management strategies. Although eHealth applications for lifestyle promotion are widely available, eHealth tailored to clients with SMI is limited [18]. Thoroughly tested eHealth for clients with SMI is even scarcer. This stresses the need to probe the possibilities of eHealth for somatic screening and lifestyle interventions [5]. To investigate the potential of eHealth, this study will evaluate the nurse-led GILL (Dutch acronym for *Gezond in Lichaam en Leefstijl*, ‘Healthy in Body and Lifestyle’) eHealth intervention, developed for clients with SMI with a complementary focus on somatic screening and lifestyle promotion [19].

For this study, GILL eHealth will be implemented in different types of healthcare facilities for clients with SMI in the Netherlands. Most clients are treated by Flexible Assertive Community Treatment (FACT) teams. These teams are multidisciplinary and focus on rehabilitation, illness and symptom management, functioning in daily life, and providing recovery support [20, 21]. Clients treated by FACT teams often live independently or in assisted facilities [22]. Another relatively small portion of clients live in inpatient facilities and receive treatment and support there. These clients have significantly higher mortality rates related to physical comorbidity than clients treated by FACT teams [1]. Systematic somatic screening and lifestyle promotion is incorporated in international guidelines for all clients with SMI [23], regardless of their living situation.

As it is unknown whether clients benefit from the GILL eHealth programme in improving physical health and lifestyle behaviours, there is an urgent need to evaluate this eHealth intervention. Therefore, the first aim of this study is to evaluate the effectiveness of the GILL eHealth intervention in clients with SMI, compared to care as usual. Special focus will be on cardiometabolic health risks and diseases of clients with SMI. Second, a process evaluation will be conducted to assess the implementation and the experiences of both clients and nurses with GILL eHealth.

## Methods

### Study design

A multicentre, cluster-randomised controlled trial (RCT) with an embedded process evaluation on the execution of the GILL eHealth intervention.

### Setting

Approximately 20 mental healthcare facilities (FACT teams, assisted facilities and long-stay wards of mental hospitals) will participate in this study. The total amount of teams will depend on the recruitment rate. When the enrolment of participants is falls short, additional teams will be added. Teams will be recruited throughout different regions across the Netherlands. They will first receive brief information about the study. If they are interested in participating, an additional presentation for all team members will be provided. After this presentation the teams can decide whether they will participate in the study. An overview of participating healthcare facilities will be available at the webpage of the trial registration.

### Participants

The study population will consist of adults with SMI, according to the definition of Delespaul [24], age 18 to 65, a body mass index (BMI) of 27 or higher, and ability and willingness to participate in the intervention. All participants will sign informed consent before participating in the study. Clients are not eligible to participate if they meet any of the following criteria: contraindications due to acute psychiatric crisis or severe physical diseases (assessed by the treating physician/psychiatrist); being pregnant or breastfeeding at the time of inclusion; cognitive impairment that interferes with the ability to provide informed consent, complete study questionnaires, or participate in the intervention; lack of internet access or inability to communicate in the Dutch language.

### Randomisation

Randomisation will take place at the team level, to avoid contamination between the intervention and control groups. Before the inclusion of clients, participating teams will be randomly assigned to the GILL eHealth intervention or the control group providing care as usual. This procedure will be performed by a statistician blinded to the characteristics of the teams using a computer-generated list of numbers. To optimise comparability of the subgroups, treatment settings (FACT, assisted housing, long-stay wards) will be matched before randomisation. Blinding of clients and participating teams is not possible due to the nature of the intervention.

### Recruitment

**Fig. 1:**
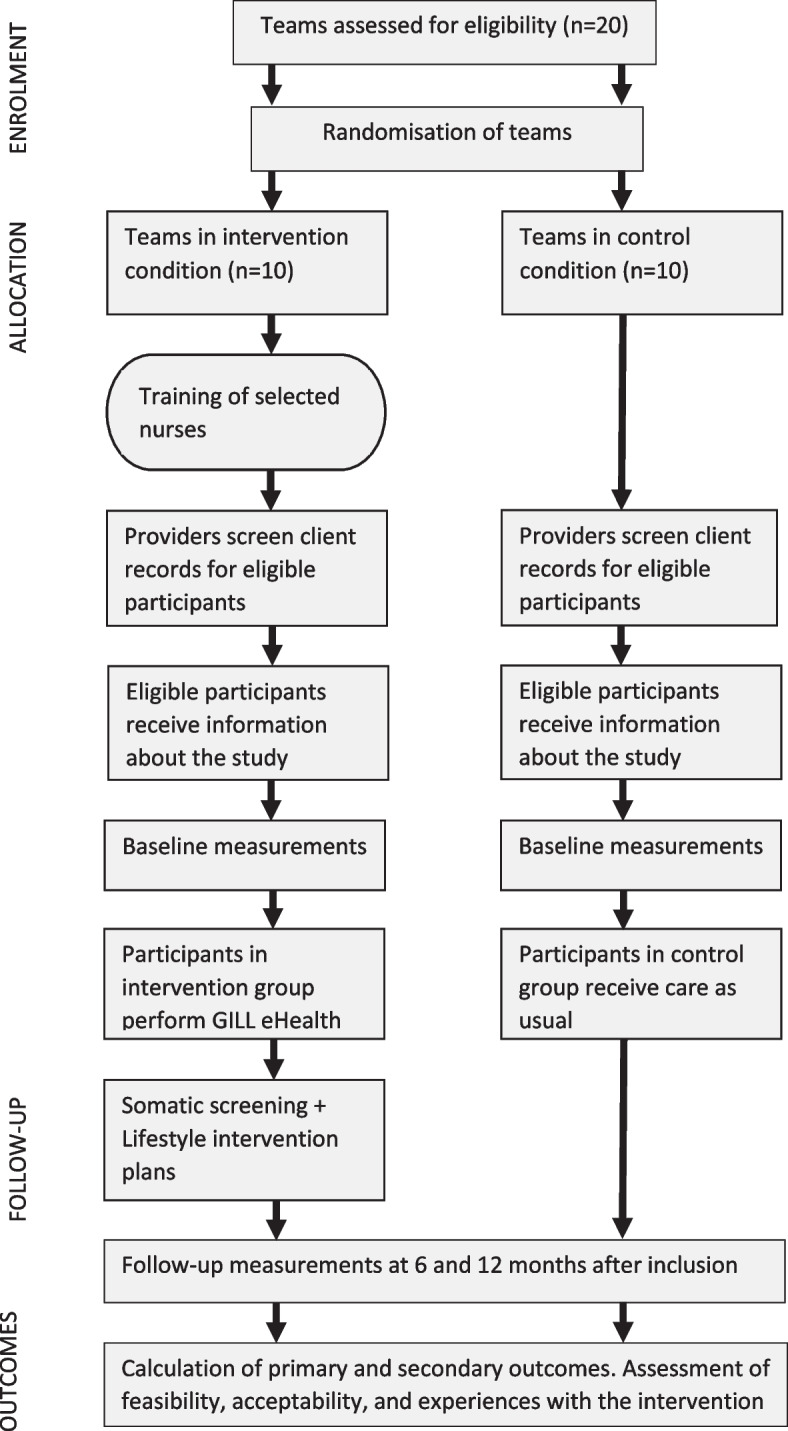
Overview of the study design and participant flow

To recruit the eligible clients, teams will compose a list of clients who meet the inclusion and exclusion criteria. Eligible clients will be asked whether they are interested in participating in this study. After giving permission, clients will be approached by the research team and further information about the study will be provided. Participants will be asked to sign an informed consent form before the first study measurements, and are given an study number to maintain confidentiality. An overview of the study design and client flow is presented in Fig. 1. Expected date of first enrolment is estimated in November 2023.

### GILL eHealth intervention

The GILL eHealth intervention was developed by two nurse practitioners and a professor of mental health nursing. The content is developed in accordance with the 2015 national guidelines on somatic screening and lifestyle intervention for clients with SMI [19]. The total refinement of GILL eHealth took approximately 10 years, developing and testing different predecessors. One predecessor, the Traffic Light Method for lifestyle promotion, showed an improvement of physical health outcomes in a pilot study [25]. GILL eHealth will be available on the platform of eHealth provider Minddistrict, ensuring appropriate technical support for the eHealth users. The intervention is both android- and iOS-compatible. GILL eHealth is a practice- and evidence-based tool that integrates somatic screening and lifestyle promotion, resulting in a personalised treatment and lifestyle plan. The intervention consists of two modules, OurGILL (somatic screening) and MyGILL (lifestyle promotion).

#### OurGILL

The OurGILL module focuses on systematic (i.e. in design, frequency, and imbedding in daily care) and comprehensive somatic screening, and promotes the prevention, early recognition, and treatment of physical problems. It aims to assess clients’ psychiatric condition, physical symptoms and complaints, medication use and side effects, specific measurements (e.g. BMI, waist circumference, laboratory measurements), and observations (e.g. extrapyramidal movement disorders). OurGILL provides an overview of physical abnormalities, which forms the basis for a personalised somatic treatment plan.

#### MyGILL

The MyGILL module is aimed at lifestyle behaviours, including nutrition, physical activity, sleep, relaxation, substance use and addiction, personal hygiene, smoking, sex life, and social support. The result is an overview of the client’s performance in these lifestyle areas. This facilitates drawing up a personalised lifestyle plan based on the client’s preferences and capabilities. The client will be guided towards the SMART formulation of lifestyle goals (specific, measurable, acceptable, realistic, and time-bound). The MyGILL module is available on both the internet platform and a mobile device application.

The two modules, OurGILL and MyGILL, are complementary and can be executed simultaneously. Implementing the two modules costs at least two hours, depending on the client’s technical abilities and self-management skills. The coordinating nurse (mental health nurse or nurse practitioner) will have a central position as ‘care manager’ when implementing the eHealth intervention, to ensure effective coordination and continuity of care within the multidisciplinary treatment context.

### Usual care

The teams assigned to the control group will not receive any training and will provide care as usual. Clients in the usual care group will have unrestricted access to mental and general health care, but are not allowed to participate in a structured lifestyle programme for the duration of the study. National guidelines are used as frame of reference for usual care, but the implementation of usual care is currently variable between teams and mental healthcare facilities. To obtain a clear overview of the care as usual as currently delivered, a questionnaire will be filled out at baseline for all participating teams focusing on the execution of somatic screening and lifestyle interventions.

### Training of care managers

The GILL eHealth intervention will be conducted by trained coordinating nurses, who will perform the role of ‘care manager’ for physical health and lifestyle. Two nurses of each team in the experimental condition will receive a one-day training focused on the theoretical background of physical health care and lifestyle promotion for clients with SMI. Most of the training time is spent on practical skills for the effective provision of the screening and lifestyle intervention to the client. Special attention is paid to motivational skills, promoting clients’ commitment to the intervention, and realising their optimal use of self-management skills. Since these nurses will act as care managers, they will be trained in how to involve the client’s social network and how they can assist in supporting clients to improve their physical health. Two eLearning modules need to be completed before participating in the training.

During the one-year follow-up period feedback sessions will be organised every three months for the nurses in the experimental condition. These sessions will be supervised by the expert trainer, focusing on barriers and successes during the implementation of the intervention. The sessions aim at learning from each other. This also provides the opportunity to detect possible bottlenecks during the implementation process, for example related to personal or organisational factors that hinder execution of the intervention. The training will be given by an experienced trainer in physical health and lifestyle promotion in mental health care. The trained nurses can function as role models within their team and disseminate their gained knowledge to other team members. Besides the structured training and feedback meetings, nurses can always contact the training staff to discuss any questions or problems that may arise.

### Main study outcomes

#### Primary outcome

Primary outcome of this study is metabolic syndrome severity, and is operationalised by the Metabolic Syndrome Severity Score (MSSS) at 12 months as defined by Gurka et al. [26]. This is the primary outcome given that the included clients have overweight/obesity (BMI 27 and higher) and a significantly increased risk of metabolic abnormalities. MSSS includes the components gender, ethnicity, systolic blood pressure, waistline circumference (cm), high-density lipoprotein, triglycerides, and fasting blood glucose [26–28].

#### Secondary outcomes

Secondary clinical outcomes are weight (kg), BMI (kg/m2), diastolic blood pressure, lipid profiles (LDL, total cholesterol), and HbA1c. Participants will also perform a six-minute walk test (6MWT) to assess physical fitness [29]. The 6MWT is a reliable and valid measure of cardiovascular fitness for overweight/obese adults [30, 31] and has been previously used in clients with SMI [32].

#### Client reports

Several client reports will be used in this study. The International Physical Activity Questionnaire Short Form (IPAQ-SF) [33] will be administered to measure each participant’s general level of physical activity. IPAQ-SF is a self-report questionnaire where participants recall the number of days and minutes of vigorous activity, moderate activity, walking time, and sitting time over the past 7 days. IPAQ-SF has been used extensively in other psychiatric populations and has acceptable validity and reliability [34, 35].

Perceived satisfaction with physical health, physical activity, and healthy eating will be measured using a numeric rating scale (NRS), with a score ranging from 0 to 10. The self-assessment concerns the past four weeks and has proven feasible [36].

Quality of life is measured using the 12-item Short-Form survey (SF-12). This is a generic, reliable and validated instrument containing 12 items derived from the 36-item Short-Form survey (SF-36) [37]. Physical and mental quality of life will be measured using the physical and mental component summary of the SF-12.

Recovery is assessed with the Questionnaire about Processes of Recovery (QPR) [38]. The QPR is a self-report 15-item questionnaire with a score range of 0–60 (QPR total) with good internal consistency and test–retest reliability properties [39]. In this study the QPR total, intrapersonal, and interpersonal scores for participants will be calculated.

Health-related self-efficacy is measured by the Patient Activation Measure (PAM-13), a reliable questionnaire containing 13 items derived from the original PAM-22 [40]. The questionnaire assesses participants’ self-reported knowledge, skills, and confidence for health-related self-efficacy. Personal views on physical and mental health, nutritional status, physical activity status, and sleep will be asked by giving a score from fully disagree to fully agree.

#### Clinician-rated

Psychosocial functioning will be measured with the Health of the Nation Outcome Scale (HoNOS) [41]. The HoNOS is an instrument comprising 12 items on four domains (behavioural problems, organic problems, psychological symptoms, social problems), each item ranging from 0 (no problems) to 4 (severe problems). Total psychosocial functioning will be indicated by the sum of all items. The scale shows good psychometric properties [41] and is used in the large majority of lifestyle programmes among severe mentally ill residential clients [42].

#### Demographics

Demographic and clinical data (i.e., age, gender, ethnicity, marital status, educational level, employment status, psychiatric diagnoses, diagnoses of physical diseases, current smoking status) will be obtained at baseline to describe our sample, to control for possible confounders, to determine the MSSS, and to analyse the effects of sociodemographic situations on the results of the intervention.

#### Utilisation of health care

Utilisation of the medical health care will be measured by the Treatment Inventory of Costs in Clients with psychiatric disorders (TIC-P) [43]. TIC-P is a validated questionnaire designed for self-report in adult clients with a mental disorder. It is a generic questionnaire, which means that the items are not related to one specific disease in mental health. The TIC-P items to be used include 20 structured questions on the volume of medical costs, e.g., ambulatory services, private practice, and general practitioner.

For an overview of the outcome measurements, instruments, and data collection schedule during the GILL study period, see Table 1.

**Table 1:** Outcome measurements, instruments, and data collection schedule of the GILL e-health intervention

	**Baseline**	**6**	**12**
**SOMATIC HEALTH**			
Metabolic syndrome severity^a^	x	x	x
Waistline circumference (cm)	x	x	x
Blood pressure (mm/hg)	x	x	x
Lipid profiles^b^	x	x	x
Fasting blood glucose (mmol/l)	x	x	x
BMI (kg/m^2^)	x	x	x
Physical fitness (6-min walking test)	x		x
**CLIENT REPORTS**			
Physical activity (IPAQ-SF)	x	x	x
Perceived lifestyle behaviours (NRS)	x	x	x
Quality of life (SF12)	x	x	x
Recovery (QPR)	x	x	x
Health-related self-efficacy (PAM-13)	x	x	x
**CLINICIAN-RATED**			
Psychosocial functioning (HoNOS)	x	x	x
**DEMOGRAPHICS**^c^			
Age, gender, ethnicity	x		
Marital status	x		
Educational level	x		
Employment status	x		
Current psychiatric diagnoses	x		
Diagnoses of somatic diseases	x		x
Current smoking status	x	x	x
Number of years receiving mental care	x		
**COSTS**			
Healthcare utilisation (TIC-P)^d^	x	x	x
Medication use	x		x
**OTHER**			
Compliance with group sessions		Weekly /monthly	
Adverse event reporting		Ongoing collection	

^a^Also employs the components sex, age, and ethnicity

^b^Cholesterol: high-density-lipoprotein, low-density-lipoprotein, total and triglycerides (mmol/L)

^c^Based on client medical records

^d^TIC-P, measured at baseline and at 3, 6, 9 and 12 months

#### Process evaluation

This study also includes a qualitative process evaluation at the client and nurse level. Barriers and facilitators for effective use and implementation of GILL eHealth will be investigated. At the client level the goal is to understand participants’ experiences and perceptions with GILL eHealth, their responses to the different elements of the intervention, and their appreciation of the nurses’ support and coaching. At the nurse level the goal is to examine their experiences and perceptions with GILL eHealth and understand its feasibility and acceptability.

To organise the semi-structured interviews, a topic guide will be developed based on study aims, the content and procedures of the GILL eHealth intervention, clients’ and nurses’ feedback during the rollout of the intervention, and the observations during the feedback meetings. The interviews will be conducted with 15 clients and 15 trained nurses. Other input for the process evaluation will come from an implementation group formed at the start of the project, to enhance the implementation and dissemination of the intervention. The group will consist of representatives of clients and family members, nurses, educators/teachers, and managers. They will provide input in at least three formal meetings on multiple aspects of implementation, such as the content of training and implementation strategies during the study.

The results of the process evaluation will be translated into recommendations for the implementation guide, developed during the final stage of the project. The implementation guide will be developed using the framework of Fleuren et al. [44] and will include an evidence-based multifaceted strategy that matches the facilitators and barriers identified in the process evaluation.

#### Sample size

This RCT is powered to detect a mean difference of at least 0.40 in the metabolic syndrome severity Z score across 1 year between the intervention and control group. The mean difference in the metabolic syndrome severity Z score is estimated on the basis of earlier intervention studies [27, 45]. With a power set at 0.80, and an alpha of 0.05, two groups of 98 patients with SMI are needed in each group. Assuming an inclusion of 10 clusters (teams) within each arm, and assuming an ICC of 0.01 implies that 220 patients are required. Assuming a dropout rate of 15% [46, 47] and testing the intervention effect using two samples t-test, implies that we need to recruit a total of 258 patients. However, testing of the intervention effect by use of linear mixed models is likely to reduce bias and increase power [48].

### Statistical analysis

Baseline data will be presented comparing the two treatment groups. Both intention-to-treat and per-protocol analysis will be conducted. To test the hypothesis that the tailored eHealth intervention will result in improved metabolic syndrome severity in SMI clients compared to usual care, linear mixed models will be used. The obtained betas describe the reduction in metabolic syndrome (Mets) Z score in the intervention group relative to the control group. Mixed-model analyses take the dependence of the repeated measurements into account, while using the maximum amount of information that is present in the data [49]. The main analyses will consist of fully corrected models. These models will be corrected for baseline values of the respective outcome plus include the covariates gender, age, and any other possible confounding variables on which the treatment groups differed at baseline. Also, ANCOVA will be used with the change of the Mets Z score (baseline – 12 months) as the outcome variable. Explanatory variables are gender, Mets Z score at baseline, and study group (intervention vs. control). The eHealth intervention succeeds if the lower mark of the two-sided 95% confidence interval is larger than zero. To test for within-group differences from baseline to the end of the intervention period, a repeated-measures ANOVA will be applied. The type-1 error is set to 5% (two-sided). For secondary outcomes, linear and logistic mixed models (depending on the outcome) will also be used to test the differences between the two groups. Missing values after 12 months will be conservatively replaced by the baseline-observation-carried-forward method to avoid an overestimation of the effect through dropouts.

### Analysis process evaluation

To objectify the process evaluation, interviews will be audio recorded, transcribed verbatim for analysis. MAXQDA software will be used for coding and structuring themes, following methodology of thematic analysis [50]. To systematically evaluate aspects of implementation, questions based on the RE-AIM model [51] will be added. RE-AIM assesses five dimensions of the implementation: reach, effectiveness, adoption, implementation, and maintenance. The process of data collection and analysis is iterative, meaning that the researchers will start data analysis after the first interviews to further explore and validate emerging themes in the next interviews.

## Discussion

Limited eHealth-supported lifestyle interventions are available for clients with SMI, tailored to their specific needs and characteristics. However, given the overall burden of poor physical health on clients with SMI [7], there is an urgent need for systematic somatic screening and effective lifestyle interventions. This paper presents the design of the GILL eHealth intervention. This cluster RCT will provide new information on the effects of systematic somatic screening and lifestyle promotion on physical health in clients with SMI. It also gives new insights on the use of eHealth in this population. The process evaluation will lead to the development of an implementation guide. This might provide additional information on the implementation of eHealth programmes, which could be used for broad implementation of GILL eHealth and future eHealth modules.

A strength of this study is its pragmatic design. The study will be conducted in mental health teams and implemented as part of essential care. In this way clients, treatments, and procedures resemble daily clinical practice. This increases the generalisability of the study outcomes. Another way the generalisability is enhanced is by including clients with SMI who receive care within different treatment settings. This limits the treatment setting as influencing factor on effectiveness and implementation. An additional strength is the quality of the GILL eHealth intervention. GILL eHealth is the result of a long-term development trajectory and is adapted to the preferences of clients and nurses. The intervention is based on current national and international guidelines [19]. It is therefore a realistic assumption that the GILL eHealth intervention will be more effective than usual care in improving physical health and lifestyle behaviours.

The GILL eHealth intervention does not allow for blinding of participants and nurses for the study. However, this reflects the daily practice and matches the pragmatic study design. Most important is to prevent contamination between the two study conditions – therefore the cluster-randomised design will be applied, in which the nurses working in the usual care teams will not receive training.

The underlying reasons that stall the implementation of systematic somatic screening and lifestyle interventions could become a limitation in this study. Currently only 17% of clients with a psychotic disorder in the Netherlands receive systematic somatic screening [52], even though it is considered essential care. Nurses have a crucial role in implementing somatic screening and lifestyle plans, yet experience this as challenging because they feel they lack skills and sufficient tools to perform the tasks [53]. Additionally, it is not always clear which tasks are the responsibility of nurses or nurse practitioners, and how multidisciplinary collaboration within and outside mental health care can be organised most effectively [54–56]. For this study an implementation group will be formed, to enhance implementation and dissemination. The nurses will also be trained in providing the eHealth, fulfilling their reported needs for training [57, 58]. GILL eHealth will offer structure in the somatic screening, supporting nurses in performing their tasks as care manager and giving a clear overview of the tasks involved.

Overall, this study will yield important and relevant information on the use of eHealth modules for improving physical health and lifestyle behaviours. The GILL eHealth module can become a clinically relevant tool when proven effective. This study will also provide insight into the barriers and facilitators for effective implementation. The first study results are expected in 2025.

## Data Availability

This study will use a data-management infrastructure that meets current guidelines and regulations. After the final publication, the protocol, all data, and the statistical code will be available upon request.

## Abbreviations

SMI: Severe mental illness
GILL: *Gezond in Lichaam en Leefstijl* (Healthy in Body and Lifestyle)
FACT: Flexible Assertive Community Treatment
RCT: Randomised controlled trial
BMI: Body mass index
SMART: Specific, measurable, acceptable, realistic, and time-bound
MSSS: Metabolic Syndrome Severity Score
6MWT: Six-minute walk test
IPAQ-SF: International Physical Activity Questionnaire Short Form
NRS: Numeric rating scale
SF-12: 12-Item Short-Form survey
QPR: Questionnaire about Processes of Recovery
HoNOS: Health of the Nation Outcome Scale
PAM-13: Patient Activation Measure
TIC-P: Treatment Inventory of Costs in Clients with psychiatric disorders
Mets: Metabolic syndrome
RE-AIM: Reach, effectiveness, adoption, implementation, and maintenance
